# Supplementary material for: Are carbon emissions trading and green financial instruments synergistic? -Comprehensive quantitative research based on content analysis
Source: PLoS One. 2024 Mar 7;19(3):e0298601. doi: 10.1371/journal.pone.0298601 (PMC10919601; doi:10.1371/journal.pone.0298601)
Supplement: S1 Data — (ZIP) [file pone.0298601.s001.zip › Data/地级市Z_F工作报告.zip!/║┌┴·╜¡/╦τ╗»╩╨/└┤╫╘╣1⁄2╓┌║┼í╛┬φ┐╦╩2╛▌═°í┐_2011.docx]

各位代表：

现在，我代表市人民政府向大会报告工作，请代表审议，并请政协委员提出意见。

　　一、2010年及“十一五”时期工作完成情况

　　 2010年，在省委、省政府和市委的领导下，我们积极推进跨越发展战略，调结构、转方式、扩总量、惠民生，攻坚克难，奋力拼搏，圆满完成了市二届人大五次会议确定的工作任务。 全市地区生产总值实现733.4亿元，同比增长14.8%；财政总收入74.2亿元，增长59.9%，地方财政收入48.6亿元，增长85.9%；新建续建投资超3000万元项目301个、超亿元项目110个，总投资364亿元，拉动城镇固定资产投资达到342亿元，增长47.4%；规模以上工业增加值102.8亿元，增长32.2%；社会消费品零售总额250.5亿元，增长18.8%；城镇居民人均可支配收入9468元，增长16.3%；农民人均纯收入6389.7元，增长46.2%，绝对额首次超过全省平均水平。全市财政总收入在全省排第五位，增速排第三位。其中，全省县市增速前十位中，我们占了三个县，望奎县增速全省第一，绥棱县第八，庆安县第十。望奎全口径财政收入和一般预算收入当年都实现翻番。绥棱、庆安、青冈地方财政收入均实现当年翻番。在全省十强县前三位中，我们占了两个，肇东市连续三年位居全省10强县之首，并作为全省唯一全国百强县位次前移到87位，安达市由第五位晋升到第三位。呈现了跨越追赶的良好势头。

　　 ——农业农村工作再登新台阶。粮食生产连续7年大丰收，总产达到269.1亿斤，提前五年实现240亿斤产能，为全省千亿斤粮食产能工程做出了突出贡献。全国产粮大县达到9个，肇东再获全国产粮大县标兵。绿色食品种植面积稳居全国市（地）之首。17座大中型水库除险加固全部完成。新组建投资3000万元农机合作社10个，农业综合机械化程度居全省前列。农民专业合作社发展到2280个。畜牧业规模经营比重达到60%，实现增加值90亿元。完成人工造林42.9万亩，是省定任务的1.28倍。新农村建设扎实推进。修建农村公路1890公里，新增自来水工程581处，又解决了20.11万人的饮水安全问题。新农合覆盖率以村为单位达到100%，参合率达到98.5%以上。争取国家财政扶贫资金5171.9万元，又有6万人实现脱贫。村级活动场所、乡村卫生院所、文体广场、清洁能源工程大量增加。内转外输劳动力112万人，创收74亿元。全市已有400个村达到省级试点村建设标准，被省委、省政府授予新农村建设先进单位。

　　 ——工业经济实力增强。新上投资超亿元产业项目75个，是去年的近2倍。17个县级以上园区集聚功能增强，望奎、青冈、绥棱三个县的项目园区被批准为省级加工业示范基地。主营业务收入超亿元企业达到52户，30户重点骨干企业对工业增加值和利税的贡献额都在50%以上。争取科技创新和技术改造资金5539万元，开发新产品58种，转化重点科技成果30项，获得国家专利184项，科技拉动作用进一步凸显。通过设立中小企业发展专项资金、助保贷款风险补偿金和争取上级专项扶持等办法，为企业解决资金3亿多元，带动企业新增投资14.3亿元。顺利完成4户企业淘汰落后产能工作，新上治污设施工业企业20多户，工业节能减排目标如期实现。全市规模以上工业实现产值330.4亿元，增长55%，利税43亿元，增长31.3%。

　　 ——商贸服务业更加活跃。扩大内需战略成效明显，市场贸易量和人均消费支出大幅增加。现代商贸物流业快速发展，新建成大型商场、超市36家，连锁经营、特许经营、物流配送、电子商务等新型流通方式不断拓展。大型物流企业发展到66家，绥化长安、顺达、安达安瑞佳、博达、望奎同源等一大批物流企业快速成长。安达物流经济创造税收近两亿元。全市兑现“家电汽车下乡”、“汽车家电以旧换新”补贴1.25亿元。进出口贸易再创新高,实现进出口贸易总额1.04亿美元，增长41.4%，其中高新技术和机电产品进出口额同比增长69.6%。利用外资成效显著，实际利用外资1.06亿美元，同比增长182%，创历史最好水平。金融、通讯、烟草、石油行业快速发展，4大行业贡献税收超两亿元，增长20%以上。全市本外币各项存款余额554.3亿元，增长17.5%。各项贷款余额301.9亿元，增长27.3%。龙江银行落户绥化，正式运营的小额贷款公司发展到17家。旅游业加速发展，旅游景区发展到32处，入选全省100个最值得去的地方两个。全市房地产建筑面积1077万平方米，建筑及房地产业贡献税收8亿元。

　　 ——民生及社会事业快速发展。全市民生及社会事业投入大幅度增加。在住房保障上，拆迁城镇棚户区262万平方米，增长63.8%，开工建设659万平方米，又有40235户城镇居民陆续住上新楼房。新配建购买廉租房5490套，发放廉租房补贴7500万元。改造农村泥草房4.56万户、倒危房2867户，已有60%以上的农民告别了泥草房。我市被评为全省棚户区和泥草房改造先进单位。在社会保障上，城镇新增就业5.4万人，涌现2500个创业带头人，带动1.12万人实现就业，妇女创业成效明显。我市被省政府命名为“省级创业试点城市”。城乡低保人数达到46.9万人，比上年增加5.8万人。北林、肇东等地建成了高标准城市养老服务中心。北林养老服务中心纳入全省首家基本养老服务体系建设试点。全市中心敬老院发展到24所，有6500多孤寡老人在幸福、和谐的大家庭中安享晚年。全市参加养老保险人员达25.5万人，新增1.9万人，并提高兑现了"五七工"、"家属工"、企业离退休人员养老金标准，全部按时足额发放。绥棱、青冈被省政府确定为农村新型养老保险试点县。医疗保险取得积极进展，工伤保险实现市级统筹。建设城镇和谐社区109个、农村精品社区30个。肇东、庆安分获全国城市和农村社区建设示范县荣誉。在城乡基础设施建设上，总投入30亿元，新建城镇道路45公里，农村公路1890公里，行政村公路硬化率达99.9%；新增园林绿地面积350万平方米、集中供热面积370万平方米。全市10个污水处理项目已完成半数以上。肇东市投资近2亿元，大力推进城市道路硬化提档、绿化升级、亮化节能改造和生态水系、湿地公园建设；海伦市投资3600万元，重点实施了"一环七通"道路改造工程，硬化街路巷道50条；望奎县完成了总投资1.5亿元的城区集中供热项目。国土资源支撑保障能力增强，建设用地供给总量居全省前列，实现地产综合收益18.7亿元。在社会事业发展上，投资4.2亿元，完成校安工程98万平方米，新建义务教育标准化学校35所，教学设施得到改善，和谐文明校园建设成效显著，高考升学率及综合排名在全省上游。投资4.61亿元，新建续建医疗机构41个。城市社区医疗卫生覆盖率达90%，农村卫生"三网四化"全面完成。启动基本药物制度改革。肇东市率先在全省实行基层药品零差价销售；望奎被评为全国农村中医药工作先进单位。文化事业全面进步。有400多件文化艺术作品在国家和省获奖。竞技体育加快发展，在省十二运会上荣获55枚金牌。全市新建各类全民健身工程178处。人口计生工作走在全省前列，自然增长率控制在2.94‰以内。城乡数字电视加快推进。平安建设力度加大。坚持严打高压态势，治安防控效能提高，数字监控系统加快普及；食品药品监管工作加强；安全生产总体态势良好；大量信访问题得到有效解决，民情在线系统在全市开通。——市本级实现跨越发展。坚持解放思想、创新发展，大力实施前三年用城市开发建设拉动增长，后两年用招商上项目拉动增长的宏观战略。2010年，实现财政总收入18.3亿元，增速在全省13地市本级中排第五位，是2005年的4.3倍，翻了两番还多；全口径财政收入11.2亿元、地方财政收入11亿元，分别是2005年的2.9倍和2.6倍，均翻了一番还多。全新的产业体系基本建成。打造开发区产业平台，累计投入基础建设资金5亿多元，建成区面积6平方公里，引进建设产业项目89个，总投资79.4亿元，投资超亿元项目38个，仅2010年就达到17个，拉开了食品医药、机械电子、商贸物流、新型材料、光伏产业"一区五园"的产业框架。引进的12户光伏企业形成了相对完整的产业链条，总投资44.5亿元，初步具备了到2013年底创产值300亿元、利税30亿元的能力。打造北方光伏产业城规划纳入全省发展战略。城市功能更加完善。围绕建设新城区、改造老城区，重点实施了路网覆盖、棚改安居、精品绿化、服务升级"四大工程"，五年市政基础建设总投入近20亿元。建设改造街道200多条、100多公里，是"十五"期间的8.5倍，排水工程54公里，是"十五"期间的5.8倍，广大居民彻底告别"水泥路"、"扬灰路"；房地产开发总面积530万平方米，是"十五"期间的4.8倍，一片片崭新的住宅小区迅速改变了城市的陈旧面貌；五年内，市区绿化投资是"十五"期间的10倍，绿化覆盖面积达到938万平方米，新建改造人民广场、人民公园等各类广场、公园10处以上，打造精品绿化景观带、花园式校园和小区77个，更进一步增添了城市风景；安装路灯1623基，是"十五"期间的2.2倍；五年前，我们没有集中供热，去年集中供热面积达到了850万平方米，拆掉了450座大小烟囱，不但老百姓的屋子更暖了，冬季空气质量也提高了；新建改造了市医院、人民医院、中医院、职教中心、实验中学、科技文化馆、五环广场、体育公园等大型公共服务设施，新建了垃圾处理厂、污水处理厂并投入使用，水、电、通讯、电视等设施进一步健全完善，申禄达、时代广场、世纪华辰、阳光购物、大世界家居等一批大型购物场所相继落成，公共服务设施全面提档升级。改善民生成效显著。近3年来，通过多方争取投入，改善民生资金超过30亿元。拆迁棚户区167万平方米，市区2万多户居民没花一分钱就把旧房换成了新房、破房换成了好房，使一些家庭几乎一夜间就成为10万元户，百姓增加财产性收入超过20亿元；配建购买廉租房21.2万平方米，市区有4000多无房户不用拿租金就能享受到家的温暖。大力度的棚户区改造，不仅使我们的房价在全国地级城市最低，也使住房问题解决的更好，受益最大的是老百姓。积极推进机关职工团购房建设，已有220人拿到了住房钥匙。发放住房补贴、取暖补贴、物价补贴7300万元，保证了低收入群体安全过冬、欢乐过节；通过开发购买公益岗位、"三支一扶"、军转安置、考录等形式，安置就业2万多人次，有500多名大学生走上稳定工作岗位；连续三年为环卫工人涨工资，并纳入养老保险，使他们的现实收入和长远利益都有了保障；连续三年为市区机关干部和离退休人员发放津补贴，使人均津补贴达到了全省1.8万元的上限标准；高标准建成了老年人福利院，筹建了儿童福利院和流浪未成年人救助保护中心，老人和儿童权益有了更好的保障；建设了12个有一定规模的社区服务中心，服务活动更加丰富。各项改革稳步推进。靠改革激发动力、创造活力，有效解决了机关进人、财政用钱、城市公交、城市管理、服务效率、工程招投标和机关干部创业等问题，政府管理公共事务效能进一步增强。去年，我市被评为全国十大人民满意城市和中国十佳和谐可持续发展城市。在中央电视台联合国家统计局、国家邮政总局、北京大学国家发展研究院举办的全国300多个城市参加的经济生活大调查中，我市连续两年获得全国十大最具幸福感城市前两位殊荣。这是广大百姓对我们政府工作的鼓励和肯定。

　　各位代表！在我们胜利告别2010年的时候，我们深切地感到，过去的五年，是大项目建设持续推进，经济发展成效最好的五年。坚持把项目建设作为"一号工程"，用最优惠的政策吸引投资，用最扎实的作风成就项目。五年项目建设总投资近千亿元，建设投资超3000万元重点项目574个，完成投资367.9亿元，是"十五"期间的3.2倍，推动城镇固定资产投资累计达到872.5亿元，是"十五"期间的4.7倍。五年内，地区生产总值翻一番还多，人均地区生产总值突破万元大关，达到1.25万元。财政总收入、全口径财政收入和一般预算收入四年翻一番，地方财政收入五年翻两番。标志着我们已经成功走上了一条大项目带动大投入、大项目牵动大调整、大项目推动大发展的跨越追赶之路。过去的五年，是基础设施不断完善，城乡面貌变化最大的五年。坚持彻底改变城乡面貌不动摇，持续增加投入，力度之大、范围之广、影响之深，超过历史任何时期。城市建设规模总量翻番。铺装改造城镇道路442公里、排水管网571.5公里，分别是"十五"期间的3.2倍和1.84倍；棚改和商品房开发面积2070万平方米，是"十五"期间的4倍；全市城镇绿化覆盖率平均达到30%，增长了10个百分点，集中供热面积增加了1200万平方米。城镇休闲广场数量增多，市民健身有了更多的去处，每个县市都至少建有一处大型公园广场；建设了10个污水处理厂，化解了区域限批危机，城市上百年污水直排历史即将结束。新农村建设亮点纷呈。投资64亿元，建设农村公路1.79万公里，是"十五"期间的11倍，投资额和建设里程连续五年居全省之首。四通八达的乡村公路网极大地改变了农村面貌；农业基础建设显著加强，现代农业示范区建设经验在全省、全国推广；农村面貌整洁有序，农民文化生活丰富多彩。五年的建设，使我们的路畅了、楼高了、灯亮了、水清了、街绿了、房好了、广场多了。带来的是老百姓拍手叫好，心情舒畅，喜气洋洋。标志着我们倾力实施的基础设施建设工程，极大地满足了广大人民群众的愿望和要求。过去的五年，是社会各项事业协调发展，人民群众得到实惠最多的五年。坚持民生预算，舍得民生投入，广泛集聚各类资源资本投向民生事业，使城乡广大群众得到了从未有过的实惠。城镇居民可支配收入是"十五"末的1.93倍，农民人均纯收入增长了一倍多，得到的各种惠农补贴超过70亿元。大力度的棚草、危倒房改造和廉租房建设，让19.2万户城乡居民喜迁新居，增加财产性收入超过30亿元；提高城乡低保标准，累计发放低保补助41.5亿元；争取和筹集资金13.3亿元，彻底解决了国有企业历史拖欠的职工工资；多渠道开发公益性岗位2.28万个，累计新增就业30.25万人,下岗失业人员再就业27.5万人，其中安置就业困难群体就业9.46万人，安置大学生就业2.1万人。全市城镇登记失业率保持在4%左右的较低水平；城乡医疗保险、养老保险基本实现全覆盖，五项保险范围不断扩大，累计支付补偿金65.4亿元。全市医院、学校、文化体育场馆、社区、公路客运站等公共设施建设投入达到40亿元以上，社会公共服务明显改善。五年来，我们较好地解决了广大人民群众最关心、最急迫、最难解的问题。标志着我们更加关注民生，民众福祉正在迅速提高。过去的五年，是和谐社会稳步推进，发展环境改善最明显的五年。坚持保护环境就是保护生产力、改善环境就是发展生产力。在生态建设上，投资7.49亿元，如期完成松花江流域污染防治，投资7.8亿元，新上了54项工业减排项目，建设完善了20个自然保护区，为可持续发展提供了最强力支撑。在社会稳定上，投入大量人财物力，集中清理信访积案，解决了多年遗留的老大难问题；连续开展各类治安整治会战，加强软硬件建设，犯罪率明显下降，百姓安全感明显增强。在服务发展上，积极推进重点领域改革，在企业转制、事业单位转型、简化行政审批、提高办事效率，以及城市管理、财税管理、人事管理、工程招投标等方面探索出了一整套行之有效的办法。加强勤政、廉政建设，打造阳光、高效、责任政府，各级政府的公信力、执行力不断提高。标志着我们在科学理政、和谐发展上迈出了坚实的步伐。

　　各位代表！五年的巨变令人欣喜，五年的成绩来之不易。这是省委、省政府和市委正确领导的结果，是各级人大鼎力支持的结果，是各级政协组织、民主党派和无党派人士积极献计出力的结果，是全市人民共同奋斗的结果。在此，我代表市人民政府，向参与、关心、支持绥化发展的广大干部群众和各界朋友表示衷心的感谢，并致以崇高的敬意！

　　回顾总结五年的工作，我们也清醒地看到，我市经济社会发展中还存在着不少矛盾和问题。一是经济总量不足，特别是人均经济总量少，发展的速度和质量有待于进一步提高；二是农业比重大、工业相对薄弱、三产质量不高的结构性矛盾还没有得到根本解决。三是财政收入增长与刚性支出不断扩大的矛盾仍然比较突出，保障和改善民生的任务仍很艰巨。四是个别政府部门创新意识不强、创业动力不足、工作效率不高等问题还在以不同程度和方式产生消极影响。对于这些前进中的问题，我们一定高度重视，下决心解决。

二、"十二五"时期的任务目标和2011年重点工作

　　各位代表！"十二五"时期是我市全面建设小康社会的关键时期。站在新的起点，综合分析形势发展，市委二届五次全会做出了继续推进跨越发展，加快实现绥化在龙江中部崛起的战略部署。这关系到绥化未来五年在全省发展大格局中的站位和水平，关系到全市人民的福祉。我们要按照市委的部署，奋力拼搏，奋发图强，努力实现更大突破。

　　未来五年，我们要全面贯彻落实科学发展观，以科学发展为主题，以转变发展方式为主线，以"两区"建设为抓手，坚定不移地调优结构、提升产业竞争力，推进跨越发展；坚定不移地深化改革开放、加快科技进步，催生创新发展；坚定不移地搞好城乡统筹、优化生态环境，保障可持续发展；坚定不移地改善民生、扩大惠民成果，实现共享发展，为加快绥化在龙江中部崛起而努力奋斗！

　　要通过五年的科学发展，努力在提升扩大经济总量上实现更大突破。增强机遇意识、争抢意识、创业意识，勇于负重拼搏、攀高比强。全市地区生产总值、全口径财政收入、一般预算收入、城镇固定资产投资、社会消费品零售总额、外贸进出口总额分别达到1500亿元、100亿元、50亿元、1200亿元、500亿元、2亿美元以上，比"十一五"末均至少翻一番以上。创优民营经济发展环境，培养壮大民营企业家队伍，全市民营经济比重达到80%以上。努力在推进两个根本性转变上实现更大突破。转变发展方式，首先要转变思维方式和工作方式，善抓农业更善抓工业，善抓农村更善抓城镇，调强产业结构，调优城乡结构，加快实现由农业大市向工业强市的根本性转变，加快实现由城乡二元结构向城乡一体化的根本性转变，切实强化以工补农，以城带乡。巩固提升传统产业，大力发展新兴产业，工业产值达到1800亿元以上，三次产业结构调整到25：40：35，全市城镇化水平达到50%以上。努力在绿色生态环境建设上实现更大突破。发挥好环境和资源优势，大力发展生态经济，优化生态环境，建设生态文明。绿色食品产业占据全省、全国优势地位，绿化工程覆盖城乡，污染物排放大幅减少，生态文明村镇创建蔚然成风，城有乡的生态，乡有城的文明，初步形成城乡一体的绿色田园城市发展框架。努力在提高人民群众生活水平上实现更大突破。做到经济发展和改善民生同步，不断改善城乡居民生活环境，提高人民群众生活质量，努力实现城乡就业渠道更宽，社会保障能力更强，基本公共服务更好，社会文明程度更高。到2015年，城乡居民收入年均分别增长14%和12%以上。使绥化以经济实力显著增强、发展质量明显提高、人居环境日益改善、幸福指数不断提升的全新面貌崛起于龙江大地。

　　2011年，是"十二五"开局之年。我们要立足于开好局、起好步，按照市委二届五次全会部署，紧紧把握未来五年发展的战略导向，唱响中部崛起主旋律，积极应对宏观形势变化带来的挑战，抢抓机遇，埋头苦干，奋发有为，努力实现全市经济社会更好更快发展。主要预期目标是：地区生产总值增长15%以上，地方财政收入增长15%以上，规模以上工业增加值增长25%以上，城镇固定资产投资增长30%以上，社会消费品零售总额增长15%以上，城镇居民人均可支配收入和农民人均纯收入增长12%和10%以上，万元GDP能耗下降2.5%。重点做好八项工作。

　　（一）以产业项目建设为重点，大力推进工业强市

　　以工业为主导，开展好"产业项目建设年"活动，认真搞好规划，重点发展绿色食品、光伏材料、石油化工、粮食深加工、医药、纺织、机电、生物化工、新能源、新材料等10大产业。进一步加大招商引资和项目建设力度。按产业成立专项推进组，实行市级四大班子牵头抓产业项目，强化考核奖惩。立足于做大做强重点产业，进一步强化领导招商、以商招商、专业招商和定向招商，全市引进投资超亿元产业项目100个以上。积极与省里对接，争取把更多的产业项目纳入全省推进计划。对已列入全省重点推进的24个产业项目，实行严格的责任制，必保按要求的时限建成投产。对各县（市、区）上报的亿元以上工业项目，跟踪考核，跟踪问效，超额完成任务的给予奖励。提升园区载体功能。争取完成投资4亿元，进一步完善园区功能，提高承载能力。依托哈大齐工业走廊，市本级、肇东、安达三个开发区要尽快跻身全省重点园区行列。其他县市园区也要加快提档升级。立足于优势与发展共享，创新园区建设模式，打破行政区划束缚,大力发展飞地经济。对异地落户的项目，谁招商谁得税。要通过打造共同的平台，集聚和发挥好各方面的优势，由此解决好制约招商引资的环境、土地、政策等问题，迅速做大园区规模。巩固提高工业运行质效。加强对30户重点骨干企业的跟踪服务，引导扶持肇东宝迪、安达贝因美、望奎双汇、青冈龙凤、明水格林等企业加快成长为产值几十亿、以至上百亿的重量级企业。加快格林公司等拟上市企业的实质性运作，努力扩大发展空间。积极推进节能减排，支持、鼓励企业引进和开发先进适用节能技术，对已建的企业污染治理设施确保正常运行。全年工业利税要增长23%以上，万元工业增加值综合能耗下降6%。

（二）以建设现代农业为重点，大力推进新农村建设

　　要在发展现代化大农业上走在全省前列，不断提升新农村建设水平。巩固提高粮食生产能力。继续抓好粮食高产攻关，重点建设好玉米、水稻高产园区，发展设施农业、标准化农业。全市各类高产创建面积达到1000万亩以上。扩大土地规模经营，提高规模效益。全市粮食面积稳定在2500万亩以上，产能巩固在260亿斤以上。加强农业基础建设。突出大水利、大农机、大科技建设。完善水利建设规划，加大国家水利政策争取力度。加强地表水、地下水资源的科学开发利用，增打抗旱大井2500眼，增修各类蓄水塘坝1500处以上，抓好小型水库除险加固和阁山水库前期准备。发展壮大农机合作社和农机大户，深入搞好场县共建，推进农业生产全过程、多领域机械化。强化科技支撑。农业良种应用、生物、栽培、节水、纳米和病虫害防治等先进技术要得到大面积的推广使用，对业绩突出的农技推广人员和示范户给予补助和奖励。拓宽农民增收渠道。提高种粮农民收入。引导农民扩大优良水稻、玉米种植，发展优质大豆。提高特色种植收入。支持农民扩大蔬菜、杂粮、林果、花卉、苗木、食用菌、饲草等高效经济作物种植，大搞棚室生产，大搞绿色食品生产，尽快释放"种一亩顶十亩"的倍增效应。特色经济作物面积扩大到200万亩以上。提高畜牧业收入。全市奶牛、生猪、肉蛋鸡等规模化、标准化养殖大场大户发展到500个以上，畜牧业增加值突破100亿元。提高农产品加工增值收入。以寒地黑土品牌为牵动，继续在农产品精深加工上努力，着力打造"大粮食经济"、"大厨房经济"，把增值收益留在本地，努力变"粮山乳海"为"金山银海"。提高农民工资性收入。大力发展劳务产业，打造劳务品牌。深入推进新农村建设。突出前瞻性，完善新农村建设规划，突出抓好中心村镇建设。大力实施整村整乡推进，促进工业向园区集中、自然屯向中心村集中、边远乡村向中心镇集中。拓宽和新建农村公路1000公里，加快村屯内道路硬化速度。新增安全饮水工程450处，新增大型沼气项目3处以上，新增村屯四旁绿化面积1万亩以上。完成农村造林面积15万亩以上。加强医疗、文化等公益设施建设，进一步改善农民生活条件。推广绥棱经验，抓好弱势村转化，完成好第五批整村扶贫开发任务。加快发展乡镇经济。站在城乡统筹的战略高度谋划乡镇经济发展，落实好市委出台的促进乡镇经济发展的实施意见。推广安达乡镇财政增收和肇东农村"三资"管理的经验，注意从体制、机制上调动乡村两级抓项目、抓财源、抓管理的积极性，加快乡村两级经济发展。

　　（三）以现代物流业为重点，大力推进第三产业优化升级

　　围绕推动第三产业优化升级，扩大商贸总量。突出发展现代物流业。重点建设好安达石化、兰西亚麻纺织、海伦大豆、庆安绿色食品四大专业物流中心和申禄达建材市场、欧亚达农机大市场、田地源农资市场、绥化市果蔬批发大市场和北方汽车城五大商品集散中心，增强集聚扩散功能。注重引进、支持跨区域大型物流公司发展，重点打造绥化、安达、青冈、海伦等区域性物流节点。大力扶持安瑞佳、博达等第三方物流企业发展，加快壮大物流群体。全市物流经济对财政贡献份额要达10%以上。积极推动消费升级。改善居民消费条件，发展物流配送、特许经营、连锁经营等现代流通方式，积极引进国内外大型超市和商场，满足多层次消费需求。打造购房、购车、旅游、文化、餐饮、娱乐健身等消费热点，提高消费层次。加强农村消费网络建设。继续推进"万村千乡市场"和"新网"工程，开展好"家电汽车下乡"和"汽车家电以旧换新"等活动，进一步激活农村市场。大力发展现代服务业。完善政策扶持措施，重点发展现代金融保险、信息服务、外包服务、文化创意、商务中介等面向生产的新兴服务业，改造提升商饮服等面向生活的传统服务业，大力发展社区服务业。积极发展特色旅游业。完善政策调控，促进全市房地产业稳健发展。抓好外资外贸工作。调整优化出口贸易结构，扶持引导企业继续扩大粮食精深加工和地方特色产品出口。帮助企业直接开拓国际市场，变间接出口为直接出口。加强外资项目引进，全市新上外资项目力争达到5个以上，合同利用外资1.5亿美元以上。全年进出口贸易总额增长15%以上。

　　（四）以"五镇"为重点，大力推进小城镇发展

　　围绕打造城乡一体的绿色田园城市，重点抓好城关镇、建制镇、中心镇、民族镇、路边镇建设。全市城镇基础设施建设投入比上年增长15%以上，省级试点小城镇建设全面高标准启动，力争城镇化率提高2个百分点。进一步加快县级城镇发展。对九个城关镇继续在完善功能、扩大规模、增强可持续发展能力上狠下功夫。继续搞好道路、给排水、供热、污水处理和燃气等项工程建设。高品位搞好绿化，创造条件打造滨水景观，增添城市灵韵。年内建成区和主要出城口的裸土面要全部绿化、硬化，所有巷道基本硬化。借鉴肇东、青冈、绥棱、望奎、明水等地做法，结合棚户区改造和城市开发，着力建设一批标志性、急需性、长远性的公共服务设施。积极推进安达、海伦等地的城市新区开发。建设好农村小城镇。认真抓好省里确定的12个试点小城镇。在科学规划的前提下，年内所有试点小城镇的基础建设要全面高标准启动，年末统一进行检查评比。抓住国家鼓励支持社会资源、资本向农村小城镇转移和省委、省政府大力加强小城镇建设的历史机遇，努力走好大开放、大开发、大合作的建设路子，精心策划，打包建设，吸引各路有识之士，把资本投向小城镇，把发展平台立在小城镇。对位置偏僻、资源匮乏、人口过少、发展潜力不大的乡镇，原则上逐渐并到中心镇。强化小城镇产业支撑。加强乡镇一级的招商引资和项目建设。大上产业化龙头企业吃配项目，大上有利于解决就业的劳动密集型的项目，大上具有鲜明地域和民族特色的商贸旅游项目，着力打造一批重要的工业镇、商贸镇、旅游镇和民族镇。鼓励支持乡镇之间联合创办产业集聚区，并尽可能将集聚区建在中心镇或建制镇，为小城镇开发建设提供有效支撑。

（五）以全民创业为重点，大力推进民营经济发展

　　要通过大力开展全民创业，推动地方民营经济加快成长。采取更有效的手段激励引导创业。大力弘扬创业文化，强化创业指导和培训，建立健全激励机制，进一步给荣誉、给政策、给地位、给奖励，激发各类人才的创业热情，让创业、乐业理念扎根于民众。扩大创业主体，继续积极鼓励支持机关干部离岗创业、各界精英及大学生在高中端领域创业、农民工返乡创业、退伍军人和下岗职工自主创业。拓展创业载体和平台。新建和完善一批创业基地和中小企业创业孵化基地，降低创业者的成本和风险。全市新增创业基地3个以上。结合特色产业园和专业市场建设，大力引进能够牵动集群式创业的龙头企业。加强中小企业公共服务平台建设，发展完善行业商会体系，为民营经济发展提供政策咨询、技术信息、信用评价、资金人才等项服务。推进融资平台建设，扶持壮大各类担保公司，不断提高担保能力。加大扶持力度。落实好全民创业和鼓励支持民营企业发展的各项扶持政策，支持创业者加快完成由创业到兴业的转变。实施中小企业成长工程，支持企业开展技术管理创新。加大财政投入，发挥好中小企业发展专项资金、工业企业助保贷款风险补偿金、小额贷款和创业基金的作用，缓解资金瓶颈制约。对妇女创业在小额财政贴息贷款上积极支持。为创业者提供更优质服务，做到注册零成本、服务零距离、办事零障碍。2011年，全市新增创业实体1.2万户以上，新增创业带头人3000人以上，非公经济增加值增长18%以上。

　　（六）以构建和谐为重点，大力推进民生和社会事业发展

　　继续加大投入，加大力度，努力在亲民爱民、惠民富民上取得新突破。突出抓好教育、就业和社会保障，进一步强化民生之本。在教育优先发展上，全面落实好国家和省新《纲要》，深入实施素质教育。重视发展学前教育，提高学前教育三年普及率。统筹发展义务教育，加快城乡学校布局优化调整。全年新增义务标准化学校30所。重视发展高中教育，切实提高教学质量。大力加强职业教育，加快技能型人才培养。全年中职招生8000人以上。大力改善办学条件，着力解决教师结构性短缺。在扩大就业再就业上，深入实施"就业优先"和创业带动就业战略，多渠道多形式创造更多就业机会。全年发放小额担保贷款4000万元以上，落实好税费减免、场地安排等政策，着力推动自主创业。继续开发政府公益性岗位，面向就业困难群体，强化安置就业。紧密结合产业发展和项目建设，加强岗前培训，依靠新上企业，扩大招工就业。力争新增就业5.6万人以上，城镇登记失业率控制在4.3%以内。在强化社会保障和社会救助上，继续推进"五险"统筹扩面，做到应保尽保。进一步提高企业退休人员养老保险金水平。抓好绥棱、青冈新农保试点，巩固提高新农合成果。深化低保等社会救助工作，努力提高城乡居民最低生活保障标准，加强对低收入群体的临时救助。继续推进中心敬老院建设，扶持民办养老机构加快发展，着力推动居家养老工作全面开展。农村五保对象集中供养率达到70%以上，城镇开展居家养老社区达到60%，每个中心乡镇至少建1个居家养老服务示范点。高度重视"稳价保供"。严格落实价格调控政策，降低流通成本；整合各项救助资金，有效保障特困群众基本生活水平不下降；依法做好价格监管，严厉打击违法涨价行为。突出抓好安居、医疗和文体惠民，更好地满足民生之需。在安居工程建设上，完成棚户区改造拆迁面积172万平方米，建筑面积490万平方米，配建廉租房6700套。完成农村泥草房改造46865户，重建维修倒危房11.5万平方米。要坚持依法拆迁、阳光拆迁、及时回迁，确保百姓住上新房子，搬进暖屋子。在完善医疗服务上，大力加强城乡医疗卫生机构基础建设，完善技术装备，强化城乡医疗卫生资源共享。对政府办基层医疗卫生机构全部实行基本药物制度，解决好用药贵问题。在文体惠民上，强化绥棱黑陶、海伦剪纸、兰西民俗风情、庆安版画四个文化产业基地建设。推进全市163个乡镇综合文化站建设全部达标，新建农家书屋200个。加快广播电视数字化进程。弘扬寒地黑土文化，鼓励支持群众性文化组织发展壮大，进一步活跃城乡文化生活。积极开展全民健身活动，推进竞技体育持续发展。创建10处以上校品训练基地，100处以上全民健身设施。加强人口计生工作，继续抓好"惠家工程"，人口自然增长率控制在5‰以内。加强妇女工作，落实好《妇女儿童发展规划》。突出抓好"三供两治"、城乡环境和社会稳定，切实打牢民生之基。在"三供两治"建设上，加强城镇二次供水系统和"一户一表"改造，提高供水质量和水费收缴率，促进节约用水。年内7个县（市）的城市污水处理厂要投入运行。加快推进集中供热和垃圾无害化处理项目。结合棚改和供热管线改造，同步推进燃气工程建设。在城乡环境建设上，继续加大工作力度和资金投入，深入搞好"三优"文明城创建和文明村镇建设，加大城乡环境综合整治力度，城市绿化覆盖率要达到30%以上，所有城市生活垃圾和医疗垃圾要及时无害化处理。全面推开城管电子数字化管理。在社会稳定上，全面加强大调解体系建设，市、县（市）区信访调解大厅6月底前投入使用。加强和创新社会管理，完善社会治安打防控体系和社会治安综合治理体系。加强公安基础建设，加强对人民群众合法权益的司法保护。充分发挥民情在线在畅达民意、解决民难上的作用，促进乡镇社区的稳定。强化安全生产责任制，突出抓好重点行业和重点领域安全隐患整治,加强敏感时段的安全检查，杜绝重特大事故发生。加强食品药品安全监管，确保城乡居民吃上放心食品、用上放心药品。

　　同时，抓好防震减灾、外事侨务、民族宗教、国防、双拥、人防、残联、老龄、武装、档案和地方志等工作。

（七）以增强综合实力为重点，大力推进市本级发展

　　着眼于打造新兴工业城、现代商贸城、绿色田园城，突出完善城市功能、壮大综合实力，以更大的力度推进市本级跨越发展。今年的主要预期目标是：全地方财政收入增长18%以上，规模以上工业增加值增长28%以上；开发区产值、增加值、利税三项指标增幅均在30%以上，新建续建投资超亿元项目15个以上。加速做大做强支柱产业。全力打造中国北方光伏产业城。以现有的新安、宝利为基础，继续加大引进战略投资商力度。已开工建设的太阳能电池板组件、多晶硅及切片、单晶切片、光伏线缆、逆变器等项目要力保如期投产达效；已签约的黑体纤维、光伏配套装备、铝件制品及光伏石墨、特种气体等项目要力推开工建设。同时，千方百计吸引国内外大企业、大集团投资，着力在较短时间内迅速膨胀投资规模。加快推进其他重点项目建设。确保投资9亿元的无机粉质环保纸、1亿元的白象方便面、1亿元的细胞酶制剂、1.5亿元的中豆制品等项目竣工投产。做大做强已建成的企业，千方百计提高贡献能力。培育发展粮油、果菜、农机、水产、建材、汽车等十大专业市场，着力推进华联商厦、大润发超市建设，抓好烟草物流项目建设。加快开发区铁路专线建设，力争建成使用。推进城市建设提档升级。本着生态化、特色化、现代化融为一体，高标准、高品位地做好2011-2030年城市总体规划修编及14平方公里新区开发设计。加强新城区生态轴和景观带建设，加速新城区西扩开发进度。继续加强城市基础设施建设。至少投资1亿元，新建改建道路11条，实施排水管网工程14项，对渗漏的地下给排水管线要一次性解决好。全程跟进绿化、亮化，新增绿地面积50万平方米以上，跟建路灯650盏以上，辟建改建广场、游园10处以上，为市民提供更多的休闲活动场所。推进西湖公园规划建设，启动红兴水库防渗工程。大力整治城市"三乱"，强化环境卫生管治，深入搞好"三优"文明城创建，全面提高城市的管理水平和文明素质。继续大力改善民生。深入实施安居工程，再拆迁改造棚户区43万平方米以上，建筑面积100万平方米以上，整个房地产新开工面积150万平方米以上。配建廉租房400套、2万平方米。要通过实物配租和货币补贴，使市区所有低保和低收入家庭的住房困难问题基本得到解决。把解决供水质量作为民生大事，抓好市区二次供水改造，统一规划建设区域性给水泵站，对不符合标准的小区供水设施彻底拆除，有效解决二次污染。加强物业管理。建立物业管理市场准入制度，着力解决物业项目经营主体过多、服务质量差、甚至乱收费、乱涨价问题。严格审定房地产开发企业接续物业管理，严格禁止不合格物业项目交付使用，切实维护好广大业主的切身利益。改造提升中盟热电供热能力，推进天然气项目尽早投入使用。抓好污水处理厂运营和垃圾处理厂配套建设。努力提高办学规模和水平，加快推进市职教中心晋升为职业技术学院。在开发区建设中小企业孵化基地，对入区创业者零租金提供办公用房。在新区建设一所小学和体育活动中心。建好"大调解"服务中心，启动使用人力资源和社会保障中心，调剂解决离退休干部活动中心用房，抓好儿童福利院、残疾人综合服务中心建设。

　　（八）以体制机制创新为重点，大力推进改革深化

　　重点推进十项改革：一是深化乡镇财政体制改革。从实际出发，在保持乡镇原有财政体制不变的情况下，积极推行核定基数、超收分成的双轨制管理办法。有条件的地方，可以实行县乡两级财政"分灶吃饭"，充分调动乡镇抓项目、抓财源的积极性。二是抓好"强镇扩权"试点。适当下放必要的管理职能和权限，让乡镇政府切实担负起建设小城镇的主体责任。三是深化财税收支管理改革。多渠道增加财政收入，鼓励发展总部经济、结算经济。完善部门预算管理，降低行政成本，建设节约政府。四是深化金融体制改革。加强信用担保机构建设，加快引进和发展地方商业银行，支持农村信用社做大做强，规范发展村镇银行、小额贷款公司、农村资金互助社等新兴农村金融组织。五是深入推进乡、村、屯撤并和教育资源整合。六是深化行政审批改革。继续精简审批程序，推行网上审批、电子监察、联动管理，提高工作效率。七是积极推进公立医院改革试点，结合实施基本药物制度，配套推进基层医疗卫生机构综合改革。八是深化工程招投标制度改革。对重要市政工程项目和公益事业项目，全面实行公开竞价招标，增强透明度，降低投资成本，提高投资效益。九是推进城市管理机制改革。进一步理顺城管体制，避免交叉管理，提高城管效率。加快建立数字化城管监控系统。十是统筹推进事业单位改革。同时要及时跟进动拆迁制度、物价监管、文化教育体制、粮食流通体制、资源税、社会管理等项改革，建立更有利于科学发展的体制机制。

　　完成上述任务目标，需要政府以更大的决心、更大的力度，不断加强自身建设。一要继续坚持解放思想，锐意进取，建设创新政府。树牢思路决定出路、观念决定贫富的理念。用发展的眼光、开放的思维、市场的规则指导工作，彻底摒弃动摇怀疑、妄自菲薄、无所作为的思想，坚定信心，树立恒心，努力在改革中寻求新突破，在发展中探索新经验，在开放中体现新举措，在工作中开创新局面。二要继续坚持转变作风，狠抓落实，建设高效政府。树牢实干就是能力，落实就是水平的理念。把抓落实作为根本工作来抓。少发文、少开会，多想事、多干事。把心思放在干事创业上，把本领用在促进发展上。比能力，首先要比抓落实的能力；比作风，首先要比抓落实的作风；比业绩，首先要比抓落实的业绩。坚决摒弃只说不干的不良风气。三要继续坚持心系群众，关注民生，建设服务政府。树牢民生是一切工作出发点和落脚点的理念。不论财力多么紧张、工作多么繁忙，改善民生的投入只能增不能减、关注民生的时间只能多不能少。落靠信访责任，对老百姓的事不推不拖，变上访为下访，履行义务，共创和谐。对企业的事，只能是多服务、少干预，多帮忙、少添乱，紧急问题及时办理，老大难问题主动办理，不违背政策法规的问题灵活办理，不断提搞政府服务水平。四要继续坚持依法行政，加强监督，建设法治政府。树牢法律至上的执政理念。规范行政行为，减少越位，纠正错位，填补缺位，做到严格公正文明执法。完善政务公开制度，经常性工作定期公开，阶段性工作逐段公开，临时性工作随时公开，切实保障广大人民群众的知情权、监督权、参与权。精简行政审批程序和项目，提高行政审批工作透明度。自觉接受人大及其常委会的法律监督，接受政协的民主监督，接受新闻媒体的舆论监督，接受人民群众的全面监督，推进阳光行政。五要继续坚持从严治政，严格自律，建设廉洁政府。树牢廉洁就是为政之本的理念。完善政府采购、工程招投标管理，扩大政府采购和招标范围，进一步规范财务管理，从体制机制上杜绝腐败，保证各级领导干部既要为民干事，又要廉洁干事、安全干事。加大行政监察和审计监督力度，发现问题，严肃处理。加强对公务员的教育和管理，树立正确的世界观、权力观、事业观，把住细节，管住小节，保住气节，做到防微杜渐，克己奉公，维护政府的良好形象。

　　各位代表！蓝图已经绘就，机遇催人奋进。让我们以科学发展观统领全局，在省委、省政府和市委的领导下，紧紧依靠全市人民，以舍我其谁的责任感、时不我待的紧迫感、不进则退的危机感，坚定信念、坚强意志，开拓创新，扎实工作，为实现中部崛起，建设美丽、富裕、和谐、强盛的新绥化而努力奋斗！
